# Ionotropic Gelation and Chemical Crosslinking as Tools to Obtain Gellan Gum-Based Beads with Mesalazine

**DOI:** 10.3390/pharmaceutics17050569

**Published:** 2025-04-25

**Authors:** Piotr Gadziński, Agnieszka Skotnicka, Natalia Lisiak, Ewa Totoń, Błażej Rubiś, Ewa Florek, Dariusz T. Mlynarczyk, Mirosław Szybowicz, Ewelina Nowak, Tomasz Osmałek

**Affiliations:** 1 Chair and Department of Pharmaceutical Technology, Poznan University of Medical Sciences, 3 Rokietnicka Street, 60-806 Poznań, Polandtosmalek@ump.edu.pl (T.O.); 2 Doctoral School, Poznan University of Medical Sciences, 70 Bukowska Street, 60-812 Poznań, Poland; 3 Department of Clinical Chemistry and Molecular Diagnostics, Poznan University of Medical Sciences, 3 Rokietnicka Street, 60-806 Poznan, Poland; nlisiak@ump.edu.pl (N.L.); etoton@ump.edu.pl (E.T.); blazejr@ump.edu.pl (B.R.); 4 Laboratory of Environmental Research, Department of Toxicology, Poznan University of Medical Sciences, 3 Rokietnicka Street, 60-806 Poznań, Poland; eflorek@ump.edu.pl; 5 Chair and Department of Chemical Technology of Drugs, Poznan University of Medical Sciences, Rokietnicka 3, 60-806 Poznan, Poland; mlynarczykd@ump.edu.pl; 6 Institute of Materials Research and Quantum Engineering, Faculty of Materials Engineering and Technical Physics, Poznań University of Technology, Piotrowo 3, 60-965 Poznań, Poland; miroslaw.szybowicz@put.poznan.pl (M.S.); ewelina.d.nowak@gmail.com (E.N.)

**Keywords:** gellan gum, ionotropic gelation, modified release, bioavailability, drug delivery

## Abstract

**Introduction:** Many orally administered drugs are either unstable in the acidic environment of the stomach or cause moderate to severe side effects in the upper gastrointestinal tract (GIT). These limitations can reduce therapeutic efficacy, discourage patient compliance, worsen the disease, and even contribute to the risk of cancer development. To overcome these issues, drug release often needs to be modified and targeted to the distal parts of the GIT. This is typically achieved through the use of pH-sensitive polymer coatings or incorporation into polymeric delivery systems. With this in mind, the aim of this project was to design, develop, and characterize gellan gum-based beads for colon-specific prolonged release of mesalazine, with potential application in the chemoprevention and treatment of bowel diseases. **Materials and Methods:** The dehydrated capsules were characterized using Raman spectroscopy and scanning electron microscopy. The crosslinked gellan gum was additionally evaluated for cytotoxicity. Key parameters such as pH-dependent swelling behavior, drug content, encapsulation efficiency, and drug release in simulated gastrointestinal fluids were also assessed. Furthermore, the behavior of the capsules in the gastrointestinal tract was studied in a rat model to evaluate their in vivo performance. **Results:** Significant differences in drug release profiles were observed between formulations crosslinked solely with calcium ions and those additionally crosslinked with glutaraldehyde (GA). The incorporation of GA effectively prolonged the release of mesalazine. These findings were further supported by in vivo studies conducted on Wistar rats, where the GA-crosslinked formulation demonstrated a markedly extended release compared to the formulation prepared using only ionotropic gelation. **Conclusions:** The combination of ionotropic gelation and glutaraldehyde crosslinking in gellan gum-based beads appears to be a promising strategy for achieving colon-specific prolonged release of mesalazine, facilitating targeted delivery to the distal regions of the gastrointestinal tract.

## 1. Introduction

Delivery of the active agent to the eligible part of the (GIT), its predictable and prolonged manner of release, as well as minimizing local and systemic side effects, have been a significant challenge for pharmaceutical technology and medicine for years [1,2,3]. Multiple drugs administered orally need protection against the acidic environment of the stomach or are intolerable to patients, causing mild to even severe side effects in the GIT and reducing the patients’ adherence [4,5,6]. Such serious defects in marketed drugs can reduce the commitment of patients to the therapy, resulting in a lowering of therapeutic efficiency. As the solution, the modification of drug release is proposed. Shifting of release of API to the more distal parts of the GIT is one of the solutions. It is usually achieved through the use of enteric coatings with pH-sensitive polymers or by incorporating the drug into beads, which can be polymer-based and pH-sensitive as well [7,8]. Taking into consideration many possible bowel malfunctions, there are many factors that should be addressed. Firstly, it is necessary to deliver the drug to the proper site of action to ensure its activity against the main problem. Secondly, it is also desired to extend the release to provide appropriate drug concentration in time. Pharmaceutical technology meets these expectations and proposes multiple formulations to face this challenge. Such formulations include liposomes, micro- and nanoparticles, suspensions, or emulsions [9,10,11]. Most of them contain various APIs considered as active agents against multiple inflammatory bowel diseases (IBD), ulcerative colitis (UC), colorectal cancers, or Crohn’s disease, such as steroids, mesalazine (5-ASA), antibiotics, or probiotics [12,13]. Unfortunately, the vast majority of marketed drugs are non-targeted and their efficacy can be significantly lowered because of the absorption of the API before it reaches the targeted site, acting systematically instead of locally and causing unwanted side effects. Therefore, developing a novel dosage form with desired characteristics compared to the used formulations seems to be a very current issue.

Taking the above into account, it seems that natural or natural-derived polymer-based beads manufactured using specific phenomena called ionotropic gelation (IG) which is an ability to go through gelation in contact with cations or/and chemical crosslinking can rise to the occasion and be suitable for delivering API to the distal part of the GIT. One of the most currently described natural polymers is gellan gum (GG). Its ability to undergo a rapid sol-gel transition as a result of IG can be used to form drug-loaded matrices both to change the site of drug release and to provide protection of API against the acidic environment of the stomach [14,15]. However, some papers report that gellan-based matrices prepared only by IG may have insufficient mechanical properties, which results in rapid decomposition of the bead and release of the drug before reaching the specific site [16,17]. To solve that problem, chemical crosslinking has been proposed as the modification and improvement of the 3D structure of prepared beads [18]. Among the multiple crosslinking agents used in this method, glutaraldehyde (GA) seems to be an appropriate tool to achieve the desirable features of the formulation [19,20]. GA is a 5-carbon linear dialdehyde, an oily liquid, excellently soluble in water, alcohol, and organic solvents. It provides crosslinking in two different ways: intermolecular, when crosslinking takes place among groups placed on different molecules, and intramolecular, when crosslinking takes place between groups located in the same molecule [21,22]. GA provides good improvement in the mechanical properties of various materials, and crosslinking has been the most common approach to overcome the limitations of multiple biomaterials. However, while considering its pharmaceutical use, one has to keep in mind that GA-crosslinked materials need to be examined for cell toxicity, while it was described that concentrations of GA below 8% can be regarded as non-toxic [16]. As it was mentioned before, a good candidate for colon-targeted formulation can be a nonsteroidal anti-inflammatory drug (NSAID). One of the proposed APIs for that purpose is 5-aminosalicylic acid (5-ASA), which is also known as mesalamine or mesalazine. It is a monohydroxybenzoic acid that structurally is a salicylic acid substituted by an amino group at the 5-position. 5-ASA, considered a colon-specific drug and locally active in the intestines, is generally well-tolerated. Its advantages include long-term therapy compatibility with proper safety and relatively low cost. The effectiveness of treatment can be improved by increasing the local concentration of the drug. This simultaneously decreases systemic side effects. 5-ASA after oral administration is normally rapidly absorbed in the proximal GI tract, so commonly used formulations are modified release (MR). However, commonly used MR tablets and suggested-in-literature novel dosage forms have insufficient efficiency of therapy [3,23,24]. They release some of the API before reaching the distal part of GIT. Absorbed 5-ASA cannot act locally, and what is more, it can cause unnecessary side effects.

Therefore, comprehensive data on the development of biopolymer-based beads loaded with 5-ASA for colon-specific delivery is still lacking. The proposed project aimed to fill this knowledge gap with a thorough and precise study concerning the influence of manufacturing parameters, qualitative and quantitative composition, and crosslinking process on the physicochemical and in vivo properties of the beads. The presented project employs cutting-edge approaches in pharmaceutical technology, focusing on targeted and sustained drug release to specific regions of the GIT. The proposed strategy aligns with the shift away from conventional formulations toward precision delivery systems, which can improve therapeutic outcomes and reduce systemic side effects. Prepared formulation is based on a naturally-derived polymer which offers biocompatibility, low toxicity, and biodegradability—key features in current trends in pharmaceutical technology. The project utilizes IG, a modern, efficient method, for drug-loaded matrices production combined with chemical crosslinking using GA, aiming to improve the mechanical stability and functional performance of the beads. The project supports the principles of precision and personalized medicine, aiming to deliver drugs specifically to the site of action (e.g., the colon) and minimize systemic exposure. This is a highly relevant and forward-looking approach, especially in the treatment of chronic gastrointestinal diseases like IBD, Crohn’s disease, or ulcerative colitis. Current marketed formulations of 5-ASA often suffer from premature release and poor targeting, leading to reduced therapeutic efficacy. This project addresses those gaps with a novel design that allows more efficient localized delivery, demonstrating responsiveness to unmet clinical needs and market deficiencies. Taking all the above into consideration, it can be stated that the presented work has a significant, novel influence on the state of the art of pharmaceutical technology.

## 2. Materials and Methods

### 2.1. Materials

Mesalazine (5-ASA) was purchased from Merck (Darmstadt, Germany). Tween80, gellan LA, Phytagel, Hydroxy-ethyl cellulose, and κ-Carrageenan were purchased from Sigma-Aldrich (St. Louis, MO, USA). Glutaraldehyde (Chempur, Piekary Śląskie, Poland) was purchased from POL-AURA (Dywity, Poland). Calcium chloride was purchased from Avantor Performance Materials S.A. (Gliwice, Poland). Hydrochloric acid 0.1N, pH 1.2, DILUT-IT Dissolution media concentrate (JT Baker), acetic buffer pH = 4.5 DILUT-IT Dissolution media concentrate (JT Baker), acetonitrile HPLC isocratic grade (JT Baker), and methanol HPLC isocratic grade were purchased from Witko Sp. z o.o. (Łódź, Poland). Phosphate buffer pH = 7.4 concentrate was purchased from Chempur (Piekary Śląskie, Poland). Deionized water was prepared by the Simplicity Water Purification System (Merck Millipore, Darmstadt, Germany); Ketamine, Kepro, (Woerden, the Netherlands); Xylazine, Kela (Hoogstraten, Belgium). Software used for statistical analyses and visualization: GraphPad Prism 5, (San Diego, CA, USA), Excel software version 2503 (Microsoft, Redmond, WA, USA), Statistica version 13 software, (TIBCO Software, Palo Alto, CA, USA). 

### 2.2. Methods

#### 2.2.1. Composition and Preparation of 5-ASA-Loaded Matrices

**Figure 1:**
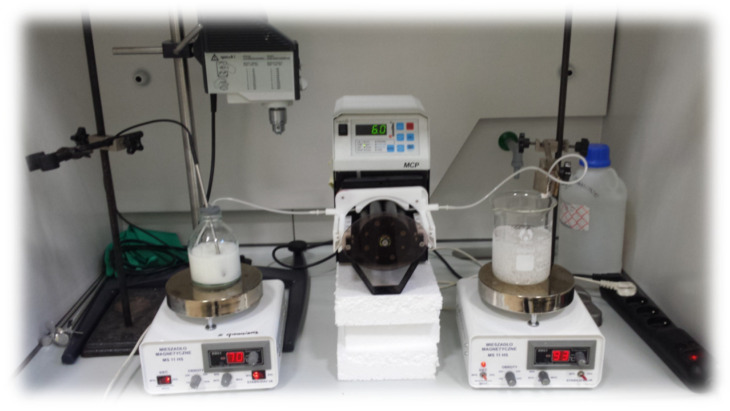
Schematic setup for droplet formulation preparation.

Matrices were obtained by ionotropic gelation or ionotropic and chemical crosslinking methods. In the first step, the mixtures containing polymer or polymer mixtures, API (active pharmaceutical ingredient), and Tween 80 as a surfactant were prepared. The percent compositions of prepared formulations are presented in Table 1. In the first step, the polymers were scattered evenly on the deionized water surface to provide proper moisture at ambient temperature and left for 15 min. All glass was protected from evaporation of water. After that time, the mixture was heated to 80 ± 2 °C and was constantly stirred during the whole process with a controlled magnetic stirrer (IKA, Staufen, Germany). After obtaining a clear solution, the temperature was decreased to 70 ± 2 °C, and the surfactant was added. After proper mixing, the given amount of 5-ASA was carefully added, and mixing was continued until a homogenous suspension was obtained. Then, prepared mixtures were pumped out of the glass with a peristaltic pump (MCP IsmatecVR, Wertheim, Germany) through a single-use syringe and needle with Luer edge (0.80 × 100 mm, Supra; Ehrhardt Medizinprodukte GmbH, Geislingen, Germany) and added dropwise to the beaker containing calcium ions (1% concentration of CaCl_2_). After 15 min (curing time), beads were collected by decantation and washed three times with distilled water. The setup for the preparation of beads is presented in Figure 1. Formulations that required an additional method of crosslinking were immediately added to another beaker containing GA solution (1% solution) at 50 ± 2 °C for 2 h under continuous stirring. After that time, the beads were washed again with distilled water to remove unreacted GA. The absence of residual components was ensured by Brady’s qualitative test (with 2,4-dinitrophenylhydrazine). The formation of a yellow precipitate after the addition of washings to the Brady’s reagent indicated the residual GA in the washings, so the beads were washed until no yellow precipitate was observed. In the next step, formulations were dried in a proper manner. Heat drying was carried out under a temperature of 45 ± 2 °C for 48 h, and chosen formulations were freeze-dried with an Epsilon 2–4 (LSCplus) (Martin Christ GmbH, Osterode am Harz, Germany) freeze dryer. The dried matrices were stored in closed polypropylene string bags at ambient temperature with limited access to light.

#### 2.2.2. Scanning Electron Microscopy (SEM)

Imaging in SEM technology was conducted using a JEOL JSM-6380LA, which was equipped with an EDS (energy dispersive spectroscopy) detector. Before examination, beads were properly dried with an appropriate method and fixed to a stand using double-sided adhesive tape. The next step was coating of the assessed beads with gold. This process was performed under a vacuum in a sputter coater for 75 s and at 15 kV to obtain the electrical conductivity. The beads were observed under the magnification of ×500 and ×1000 by a scanning electron microscope.

#### 2.2.3. Raman Spectroscopy

The non-polarized Raman spectra were acquired by an inVia Renishaw micro-Raman system (Renishaw, New Mills, UK) in the backscattering geometry. The Raman spectrum was recorded within the spectral range of 100–3200 cm^−1^. A 785 nm infrared solid-state laser served as a source of exciting light. The laser beam was precisely focused on the sample surface using a Leica 50^®^ LWD microscope with a long working distance objective (LWD) (Leica, Wetzlar, Germany) and a numerical aperture (NA) of 0.5. The laser beam had a diameter of 2 μm. Detection was carried out using an air-cooled CCD camera (Rencam) in combination with an 1800 lines/mm diffraction grating. An excitation power was fixed at no more than 10 mW to prevent any damage to the sample. Analysis was performed at room temperature (23 ± 2 °C). The Raman scattering spectra for all samples were obtained under the same conditions.

#### 2.2.4. Swelling Behavior

The dried beads were examined in case of the tendency to absorb water under various pH conditions. Three liquids with different pH values were used: phosphate buffer (pH = 7.4), acetate buffer (pH = 4.5), and 0.1 M hydrochloric acid solution (pH = 1.2). The test was performed at a temperature of 37 ± 0.5 °C. Approximately 30 ± 0.1 mg of beads were placed in beakers (25 mL) and submerged in 10 mL of the liquid (previously heated to the proper temperature). Afterward, beakers were placed onto the KS 130 Control orbital shaker (IKAÆ, Staufen, Germany) and mixed at 150 rpm. The whole system was placed in a heater with gravitational air flow (Binder, Gleisdorf, Austria). After specific time intervals (1, 2, 3, and 6 h), the beads were extracted from the surrounding liquid, blotted properly with a paper towel, and then weighed. After every measurement point, beads were placed into fresh portions of acceptor liquid. The swelling index was calculated using Equation (1):

(1) $$\%SI=\frac{W2-W1}{W1}\times 100$$

W1—weight of dried beads, W2—the weight of swollen beads at a certain point in time.

#### 2.2.5. Determination of 5-ASA Content and Loading Efficiency

Prepared formulations were thoroughly weighed in the amount matching the theoretical 10.0 mg of 5-ASA, placed into the volumetric flasks, and submerged with 25 mL of ethanol, and then mixed with the orbital shaker in the heater at 37 ± 0.5 °C overnight. After 24 h, the solution in the amount of 1 mL was extracted from the vessel, diluted three times with methanol, and then the HPLC analysis was performed using a 1200 Series LC chromatograph (Agilent, Santa Clara, CA, USA) on Xterra C18, 250 mm, 4.6 mm, 3.5 µm 125 Å chromatographic column (Waters, Milford, USA). The mobile phase was composed of phosphate buffer (pH = 6.0): methanol at a 60:40 (*v/v*) ratio. Analysis was performed under isocratic conditions with the flow rate at 1.2 mL/min. The temperature of the column was thermostatically controlled at 30 ± 0.5 °C. Measurements were performed at an analytical wavelength of 235 nm. The drug loading and drug encapsulation efficiency were calculated using Equations (2) and (3).

(2) $$Drug\;loading\left(\%\right)=\frac{amount\;of\;5-ASA\;in\;the\;beads}{mass\;of\;the\;beads}\times 100$$

(3) $$5-ASA\;encapsulation\;efficiency\left(\%\right)=\frac{actual\;5-ASA\;content\;inbeads}{theoretical\;API\;content\;in\;beads}\times 100$$

#### 2.2.6. In Vitro Drug Release Studies

In vitro drug release studies were conducted using a USP 1 apparatus (Kraemer Elektronik GmbH, Darmstadt, Germany). Tests were performed in different pH conditions, separately for pH = 1.2 and pH = 7.4, in 900 mL of acceptor fluid and at a temperature of 37 ± 0.5 °C. Approximately amount of 100 mg of beads were placed in the basket and submerged into a proper acceptor fluid. The total time of the experiment for pH = 1.2 and pH 7.4 was set at 120 and 360 min, respectively. The basket rotation speed was set to 100 rpm. At certain time points, the acceptor fluid in the amount of 1 mL was withdrawn from the vessel and replaced with a fresh portion. Withdrawn fluid was analyzed according to the HPLC assay given in the previous section.

#### 2.2.7. Microtox Acute Toxicity

The Microtox acute toxicity test was performed using the Microtox M500 model (Modern Water). The modified 81.9% screening test was used with modifications described before [25]. Briefly, after recording the bioluminescence right before the sample addition, precooled 2% sodium chloride solution (Microtox Diluent, Modern Water) was added to the *Aliivibrio fischeri* bacterial suspension, after which an appropriate amount of solid formulation was immediately added, and the mixture was mixed. Further monitoring of the bioluminescence at 5 and 15 min of the test was performed according to the protocol supplied by the manufacturer.

#### 2.2.8. Biomaterial Cytotoxicity Assessment

##### Cell Culture

The colorectal *adenocarcinoma* cell line Caco-2 (ATCC^®^ HTB-37™) and the nontumorigenic mammary epithelial cell line MCF-12A (ATCC^®^ CRL-10782™) were used as model cell lines to assess in vitro cytotoxicity of investigated beads. Caco-2 cells were cultured in Eagle’s Minimum Essential Medium (EMEM) (ATCC^®^, Manassas, VA, USA) supplemented with 1% (*v*/*v*) non-essential amino acids (Sigma-Aldrich, Steinheim, Germany) and 10% (*v*/*v*) fetal bovine serum (FBS) (Sigma-Aldrich, Steinheim, Germany). MCF-12A (ATCC^®^ CRL-10782™) cells were maintained in DMEM-F12 medium (Biowest, Lakewood Ranch, FL, USA) with the addition of hydrocortisone (0.5 µg/mL), insulin (10 µg/mL), human epidermal growth factor (hEGF) (20 ng/mL), cholera toxin (0.1 µg/mL), and 5% fetal horse serum (all from Sigma-Aldrich, Steinheim, Germany). Cells were grown to near confluence in 100 × 15 mm Falcon^®^ cell culture Petri dishes (Corning, Corning, NY, USA) at 37 °C in an atmosphere of 5% (*v*/*v*) CO_2_ and 95% (*v*/*v*) relative humidity. The absence of mycoplasma contamination was routinely verified using the Mycoplasma Stain Kit (Sigma-Aldrich, Steinheim, Germany).

##### Cytotoxicity Assay

The cytotoxicity was assessed using a standardized MTT assay, in which the cytotoxicity of agents was evaluated based on the metabolic activity of two tested cell lines. The assay was performed as previously described [26]. Cells were plated at a density of 5 × 103 cells per 100 μL of recommended growth medium on a sterile 96-well plate and cultured overnight to add extracts. The growth medium was used for performing extracts. Tested materials (F6, F9, F13, F15, F19, and F21) were dissolved in the recommended growth medium in a final concentration of 10 mg/mL. The extraction was carried out for 24 h/300 rpm at 37 °C in sterile conditions in darkness using an Eppendorf Thermomixer comfort 2.0. Tested extracts were added to both cell line cultures to obtain final concentrations: 2%, 5%, 10%, 20%, 50%, 75%, and 100% (*v/v*). The cells were exposed to the studied extracts for 24 h. After incubation, the medium with extracts was removed, and MTT reagent was added to each well (5.0 mg/mL) (Sigma-Aldrich, Germany). The cells were incubated at 37 °C for 4 h, after which 100 μL of solubilization buffer (10% SDS in 0.01 M HCl) was added. Absorbance at 570 nm was then measured using a Microplate Reader Multiscan FC (Thermo Scientific, USA), with a reference wavelength of 690 nm. Three independent experiments were conducted, each with three replicates per concentration. Relative cell viability was calculated using the formula: % viability = (mean of A570–A690 of an experimental group)/(mean of A570–A690 of the control group) × 100%. The viability of the cells was calculated with Excel software (Microsoft, Redmond, WA, USA).

##### Statistical Analysis for Cell Proliferation Assay

The obtained data were expressed as mean ± SD of at least three separate experiments. Statistical analysis was performed by one-way ANOVA (GraphPad Prism 5, San Diego, CA, USA). The threshold for significance was defined as *p* < 0.05.

#### 2.2.9. In Vivo Availability Studies

##### Ethics Committee Approval

The Ethics Committee for Animal Experiments Affairs in Poznan, Poland, authorized the study design (Approval No. 42/2022 of 10.06.2022). All laboratory animal handling and usage methods followed European Union (EU) laws under Directive 2010/63/EU on animal protection for research purposes [27]. Experiments were conducted using the “3Rs” principle (Replacement, Reduction, and Refinement) to safeguard animals. The study focused on the minimum number of animals and observation time to ensure consistent results. To improve the rigor and reproducibility of animal research, all data were collected using the ARRIVE 2.0 guidelines. Experiments were carried out in the Laboratory of Experimental Animals at Poznan University of Medical Sciences in Poland. The Laboratory of Experimental Animals is a unit listed by the Ministry of Science and Higher Education, Poland. Contractors have individual permits for planning and performing experiments.

##### Animals

The study involved 96 male rats (Rattus norvegicus), outbred, Wistar strain, 16 weeks old, weighing 400.0 ± 25 g, with a Health certificate CRL. The rats were sourced from a certified breeding facility, Charles River Laboratories, Germany. The animals were acclimated to laboratory conditions for 2 weeks before the trial began. Only male rats were used in the experiment to ensure reliable results and minimize animal usage (in line with the 3Rs principle). The conditions in the experimental box were optimal for the welfare of the animals. The animals in the experiment were kept in conditions of high cleanliness and constant, strictly defined, regulated and controlled atmospheric conditions (BMS system), i.e., at a temperature of 22 ± 2 °C, air humidity of 55 ± 10%, in the 24-h light cycle (12-h light/dark continuity) in an experimental and living room where the air exchange rate is 15 changes/h. The animals were kept in two per cage, with enrichment in the form of wooden blocks and cotton cocoons on dust-free aspen bedding (TAPVEI Aspen, Harjumaa, Estonia), which was changed at least once a week (or as needed). Detailed animal welfare inspections were carried out every day. The animals had free access to water and a balanced diet. They were fed a maintenance diet for rats, Altromin 1324 (Altromin, Lage, Germany). To ensure ethical compliance, we followed the guidelines of the Ethics Committee for Animal Experiments Affairs in Poznan, Poland, when conducting animal studies. There were no exclusions of animals during the analysis.

##### Experimental Design

Rats were randomly divided into three groups (I–III), each of 32 individuals. The first group (control) was administered bulk 5-ASA suspension in water with a gastric tube at a dose of 20 mg/kg. The second group received the examined formulation prepared by only IG, and the third experimental group was administered a formulation using both IG and GA (20 mg/kg). After the observation period, the animals underwent an autopsy. For anesthesia, rats were intramuscularly administered a solution of ketamine (90 mg/kg; Kepro, Woerden, the Netherlands) and xylazine (10 mg/kg; Kela, Hoogstraten, Belgium). Once deep anesthesia was achieved, the animals were sacrificed by cardiac blood collection at 0.5, 1, 2, 4, 8, 12, 16, and 24 h (four rats per time point, n = 4).

The blood was centrifuged at 3000 rpm for 10 min, after which the plasma was collected and stored at −80 °C for further analysis.

#### 2.2.10. Statistical Analysis

Statistical analyses were conducted using Statistica version 13 software. The results for the investigated formulations were subjected to a one-way analysis of variance (ANOVA). To compare the mean values of the analyzed parameters, post-hoc Scheffé’s test was applied. A significance level of 5% was used for all tests.

## 3. Results and Discussion

In the present study, we aimed to design, produce, and characterize gellan gum-based beads intended for colon-specific prolonged release of mesalazine. The primary objective was to evaluate the hypothesis regarding the influence of glutaraldehyde crosslinking on the properties of the beads, in comparison to traditional formulations stabilized solely by calcium ions. Additionally, gellan gum was used not only in its pure form but also in combination with other natural polymers. All formulations were prepared using the same method, differing only in specific components such as the type of additional polymer, the presence or absence of the active pharmaceutical ingredient (API), and the drying technique applied.

### 3.1. Morphology of 5-ASA-Loaded Matrices

**Figure 2:**
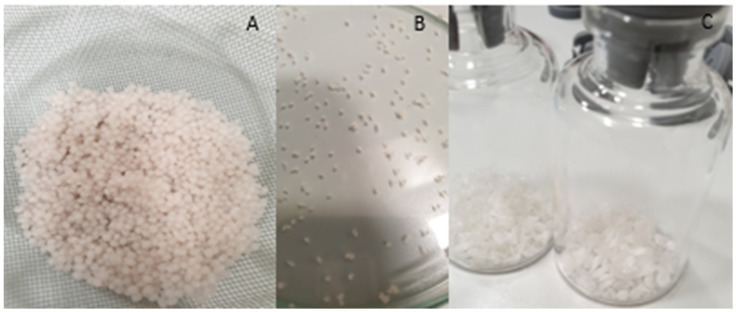
Prepared beads. (**A**) before drying, (**B**) after heat drying, (**C**) freeze-dried.

Directly after manufacturing, prepared beads were subjected to visual observations. As the morphology (structure, shape, size, and hardness) depends on a wide range of factors such as temperature of the initial suspension, concentrations of crosslinking agents and the polymer, stirring speed, height, and speed of instillation or drying technique, etc., the preparation process is complex and needs to be properly planned and optimized [27,28,29,30,31]. As shown in Figure 2, the obtained beads exhibited varying shapes. While the majority were round and spherical, some drop-like or comma-shaped beads were also observed prior to drying. The pinkish tint of the beads comes from the API, as the polymer solution is colorless, and after drying, the color of the beads is less intense [32]. After HD, the beads were hard and decreased in diameter significantly. Also, some flattening on the bottom side of the beads can be seen, which is a very common phenomena after HD as the result of water evaporation, which cause the loss of elasticity of beads [4,15]. After FD, the reduction in size was much smaller, but beads were much softer and foam-like with low density [33]. Formulations containing κC seemed to have the most spherical shape, as the beads containing HEC were the most flattened. Furthermore, the addition of κC resulted in a smoother surface of the bead compared to only the GG formulation. Such observations are consistent with previous conclusions [34].

### 3.2. Scanning Electron Microscopy (SEM)

**Figure 3:**
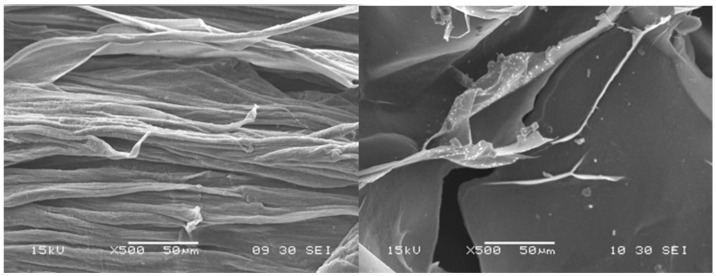
SEM micrographs at a magnification of ×500 with visible structures.

**Figure 4:**
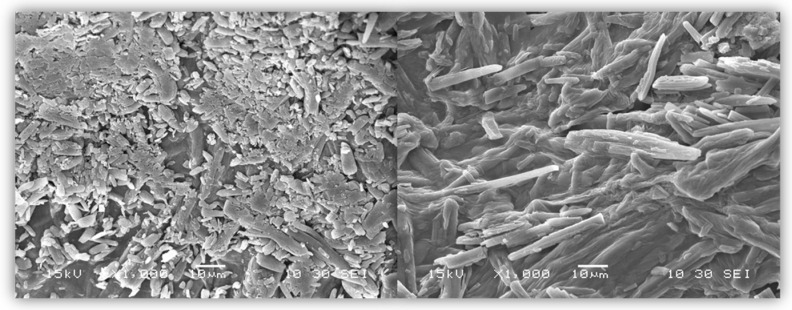
SEM micrographs at a magnification of ×1000 with visible 5-ASA crystal.

**Figure 5:**
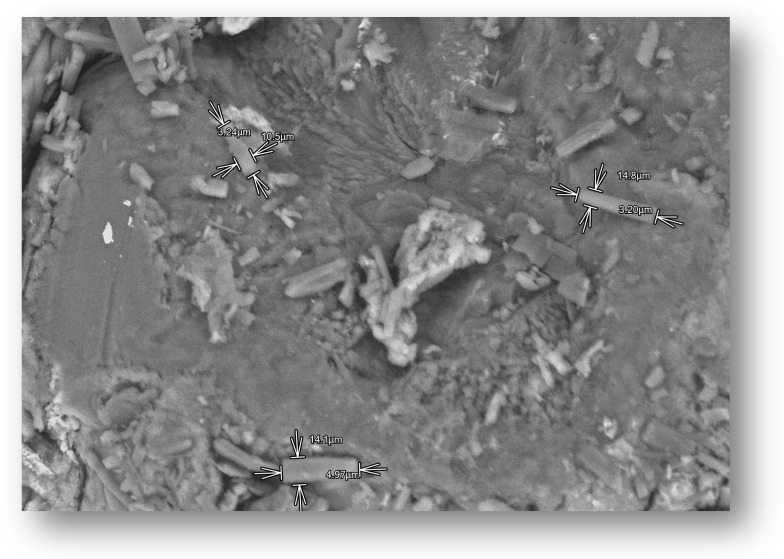
SEM micrograph of measured 5-ASA crystals with magnification ×1000.

After drying, the beads were analyzed with the use of SEM microscopy. As shown in Figure 3, Figure 4 and Figure 5, the surface of the beads was irregular, wrinkled, and most probably covered by the 5-ASA crystals. Crystals seemed to appear not only on the surface of the beads but also submerged in the matrices. On the surface, besides the API crystals, the longitudinal polymer fibers can be seen as well. No significant differences can be seen between formulations composed of different polymers. All of them created 3D structures loaded with API.

### 3.3. Raman Spectroscopy

**Figure 6:**
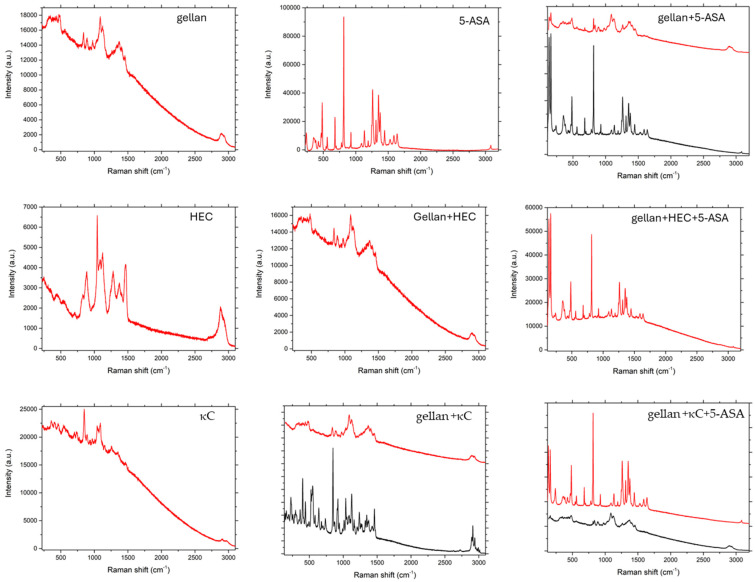
Raman spectra of pure components and their physical mixtures.

**Figure 7:**
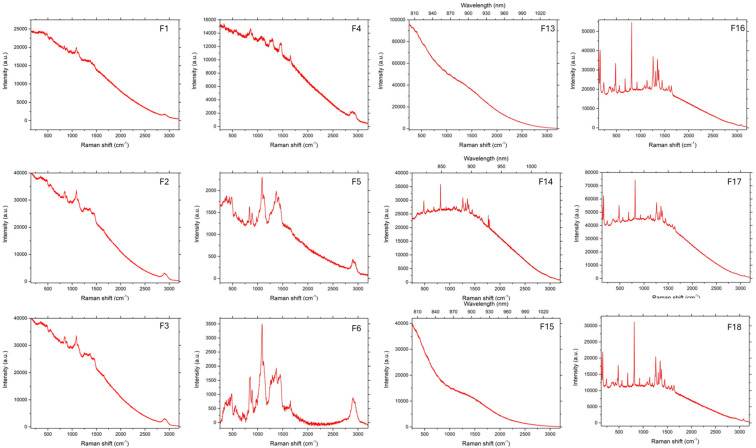
Raman spectra of beads prepared by IG.

**Figure 8:**
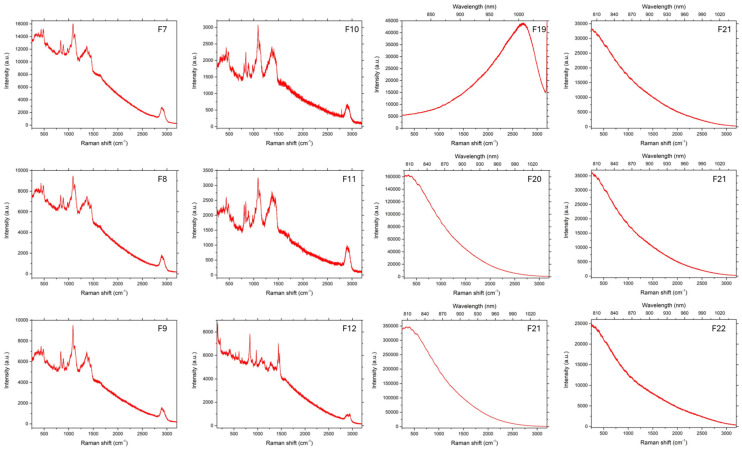
Raman spectra of beads prepared by IG and GA.

As can be seen in Figure 6, Figure 7 and Figure 8, in the case of matrices containing 5-ASA, the characteristic band for 5-ASA was observed at the wavelengths of 830 cm^−1^, 1356 cm^−1^, and 1582 cm^−1^, which are specific for the organic ring and the N-H and C-H bonds, respectively [35,36]. Their occurrence or partial fading could be the result of strong Rayleigh scattering caused by a high luminescence background; therefore Raman spectrum could not be obtained in particular cases [37]. After all, there were no significant signs of drug decomposition, as the manufacturing process was successful.

### 3.4. Swelling Behavior

**Figure 9:**
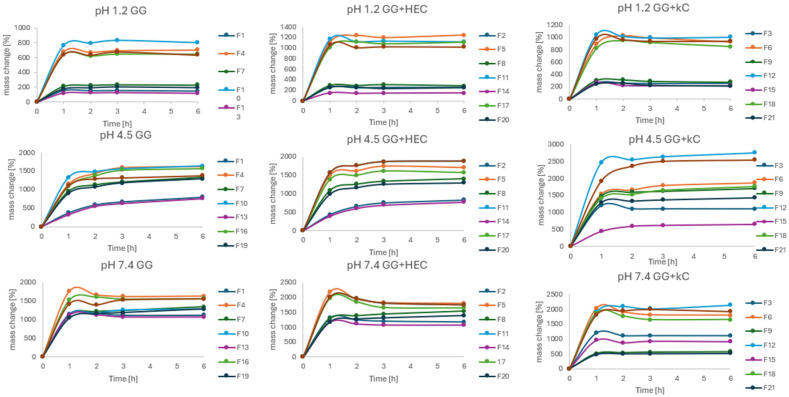
Swelling behavior of prepared beads in three different pH conditions. Error bars omitted for clarity.

The swelling behavior in various pH conditions was assessed in order to determine the influence of pH values on the tendency of the matrices to absorb water. The results of the experiment are presented in Figure 9. The trial was conducted in three different fluids differing by the pH (1.2, 4.5, and 7.4) corresponding to physiological conditions that may occur during the passage of matrices through the GIT. As can be seen, beads after drying retained the ability to absorb water in all fluids. However, rates and patterns of absorption differed significantly. The slightest changes in bead mass were observed at pH = 1.2, with the most noticeable mass growth in the first hour of the experiment. It should also be stated that the mass growth in pH = 1.2 could also be caused by water entrapment in structures and cavities of the bead surface, which could not be removed despite careful blotting. A similar effect, but with a higher rate, was observed in pH = 7.4, after significant growth in the first hour, then the mass of beads was more or less stable. At that point, it should be mentioned that swelling in alkaline solutions promotes the degradation of the beads, so mass stabilization could affect both swelling and decomposition of the beads [38]. Different from the pH = 4.5, where constant mass growth was observed even after 6 h. However, the factor having the strongest influence on water absorption seemed to be the method of drying. All the freeze-dried beads absorbed water to a significantly greater extent compared to their HD counterparts. In general, the formulations prepared by IG and GA seemed to swell less compared to others. It can also be stated that κC present in the formulation decreased the rate of swelling in all conditions (e.g., F15, F21). Such an effect of carrageenan was previously described in the literature [14].

### 3.5. Determination of 5-ASA Content and Loading Efficiency

**Figure 10:**
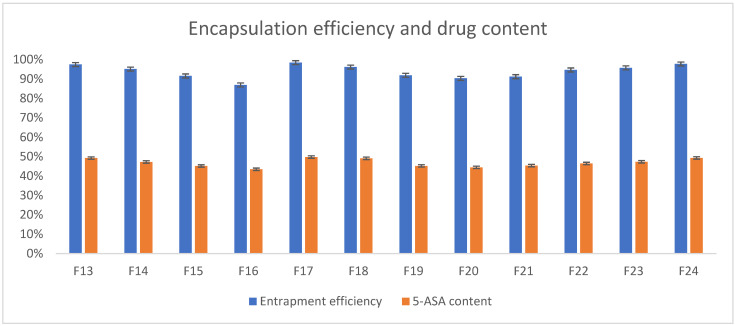
5-ASA encapsulation and content in prepared beads.

Preparation of the beads based on polymers like gellan gum and its blends resulted in obtaining matrices with a high rate of entrapment efficiency. As can be seen in Figure 10, the API almost entirely succeeded in encapsulating the beads (by a mean of 93.95%, the drug was encapsulated in all formulations), as the encapsulation efficiency varied from 86.95% to 98.45%. 5-ASA content varied from 43.47% to 49.76% of the beads’ mass (mean 46.83%). Well-prepared and optimized process of manufacturing such formulations resulted in high efficiency, which is broadly confirmed in the literature [27,39,40]. What can also be stated based on experimental results is that no significant drug decomposition was observed during the process of manufacturing.

### 3.6. In Vitro Drug Release Studies

**Figure 11:**
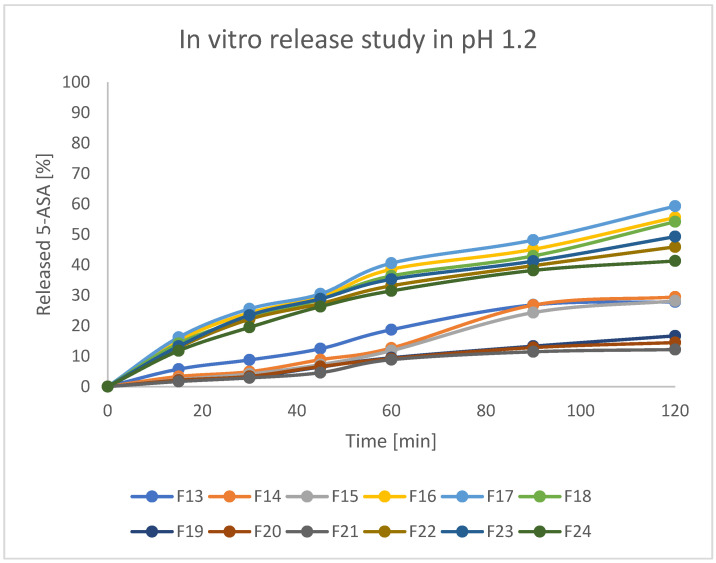
5-ASA in vitro release profile at pH = 1.2.

**Figure 12:**
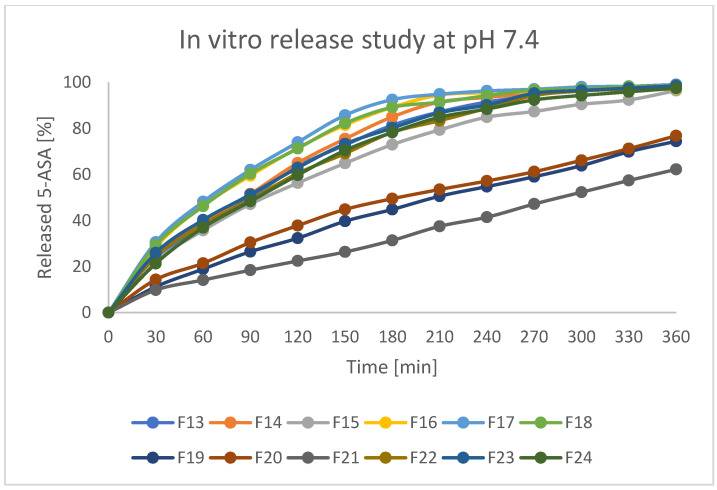
5-ASA in vitro release profile at pH = 7.4. Error bars omitted for clarity.

Assessed formulations were subjected to release tests in various pH conditions. As stated in the protocol described in European Pharmacopeia, enteric products should stay intact in an acidic environment for 2 h, and release not more than 10% of API during that time. Secondly, the experiment should be continued with higher pH values. For better clarity of the results, beads were investigated at pH = 1.2 and pH = 7.4 separately. The obtained results are presented in Figure 11 and Figure 12. As can be seen, there were significant differences in releasing the API both at pH = 1.2 and pH = 7.4. In the first step, it can be stated that formulations F19, F20, and F21 (prepared by IG and GA) released around 10–12% of incorporated 5-ASA in an acidic environment in 2 h. Formulations F13–F15 (prepared by only IG) released around 20% of API in such a time. All other formulations (mostly FD) released above 30% of API. Moreover, all the tested beads seemed to keep their shape and color during the analysis. It could indicate that prepared matrices are stable in an acidic environment and can keep the drug from releasing in the stomach under physiological conditions [41]. It is also noteworthy that the initial increase of the drug concentration in acidic acceptor fluid may be a result of washing away drug crystals from the surface of the beads (what was seen on the SEM pictures) and does not have to be the symptom of releasing the drug from the matrix of the bead. At pH = 7.4, all formulations released the drug much more intensively compared to pH = 1.2. Formulations F19–F21 (IG, GA, HD) seemed to release 5-ASA in the most prolonged manner, resulting in the release of 50–70% of API in 6 h. In the first 2 h of the analysis, formulations F19–F21 released only around 20% of the initial drug content, which suggests a significant potential in moving the remained amount of API to the distal parts. Other formulations released at least 80% of 5-ASA after 4 h of the test and kept increasing the concentration of 5-ASA in the acceptor fluid up to 100% of the expected amount. It is also noteworthy that release studies correlate with swelling behavior. Formulations characterized by a lower tendency to absorb water resulted in a lower drug release rate. This is likely due to the slower penetration of water inside the bead matrix.

To the best of our knowledge, successful studies focusing on enteric delivery of the API-containing gellan gum are quite rare. Most of them are focused on enteric coating capsules composed of gellan gum [42,43] or manufacturing gellan gum-based micro- and nanoparticles [44,45]. Based on the literature and obtained results, it can be stated that the proposed formulations can be a promising candidate for encapsulating various drugs that are expected to act in the distal part of the GIT and are worth further investigation.

### 3.7. Microtox Acute Toxicity

Besides the in vitro laboratory studies, all of the prepared formulations were subjected to the Microtox acute toxicity test, which is based on the bioluminescence decrease of *A. fischeri* bacteria upon contact with the tested sample. An interesting phenomenon was observed for the samples, where glutaraldehyde was used as the crosslinking agent, as the toxicity of the formulations was considerably higher than for other formulations, and the toxicity increased substantially after 15 min of the test, as compared to the 5-min reading. This was attributed to the leaching of the glutaraldehyde from the materials, which was reported to exhibit an antibacterial effect that might affect the Microtox test results [46].

**Figure 13:**
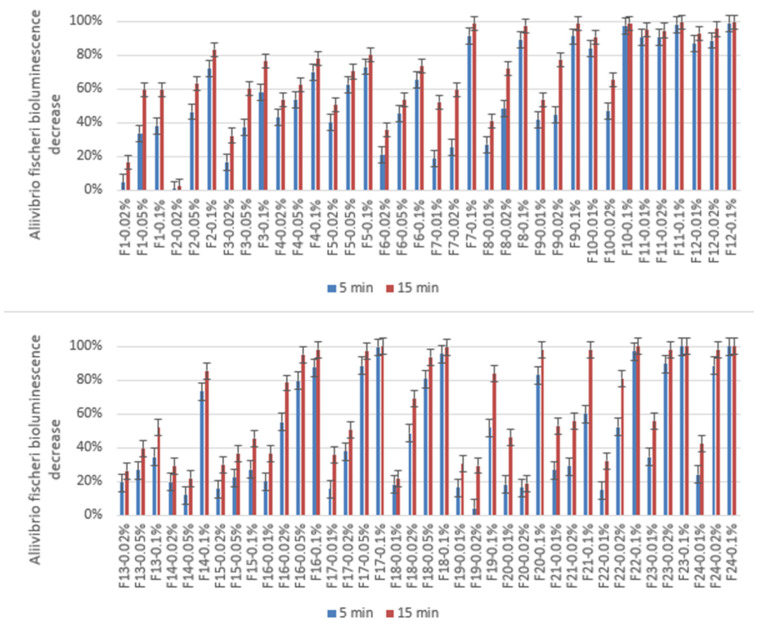
The measured *Aliivibrio fischeri* bioluminescence decrease was measured in the Microtox 81.9 Screening Test for empty formulations (F1–F12) and mesalazine-loaded formulations (F13–F24). Concentrations are given in m/m.

As the measured decrease in the bioluminescence is compared between empty and loaded formulations, it seems that the 5-ASA decreases the toxic effect of all the studied formulations, including the glutaraldehyde-containing ones. The least toxic effect of the studied drug delivery systems was exerted by F15, while F11 and F12 were too toxic to provide any useful results. The obtained results are presented in Table 2 and Figure 13.

### 3.8. Biomaterial Cytotoxicity Assessment

As the 5-ASA reportedly has antiproliferative and preventive properties in case of colorectal cancer, the MTT assay was performed to evaluate the cytotoxicity of compounds on the colon cancer cell line, Caco-2, and non-cancerous MCF-12A cells (to verify compound specificity) [46,47].

**Figure 14:**
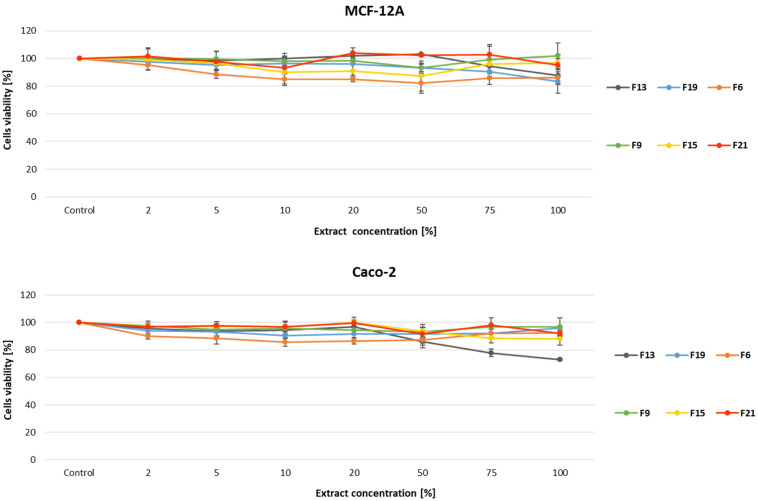
Cell viability assessment. Cells were subjected to tested extracts at concentrations: 2%, 5%, 10%, 20%, 50%, 75%, and 100% (*v*/*v*) for 24 h, followed by an MTT test. Three separate experiments were performed, with three repeats for each concentration. Data show mean values ± SD of at least three separate experiments.

Both cell lines were treated for 24 h with compounds containing gellan gum and κC based on previously obtained results and selected as the most promising formulations (F13, F19, F6, F9, F15, and F21) in the concentration range of extract 2–100%. The obtained results are presented as a percentage of the untreated control cells (Figure 14).

In this experiment, only the F13 compound in the highest extract concentration (100%) decreased the viability of Caco-2 cells (up to 25%), however, the rest of the studied compounds did not reveal significant cytotoxicity against colon cancer, Caco-2 cell line, and non-cancerous, MCF-12A cells.

In accordance with ISO10993, the cytotoxicity of a compound is acknowledged as significant only when the cell survival is lower than 70% compared to the control sample.

### 3.9. In Vivo Availability Studies

**Figure 15:**
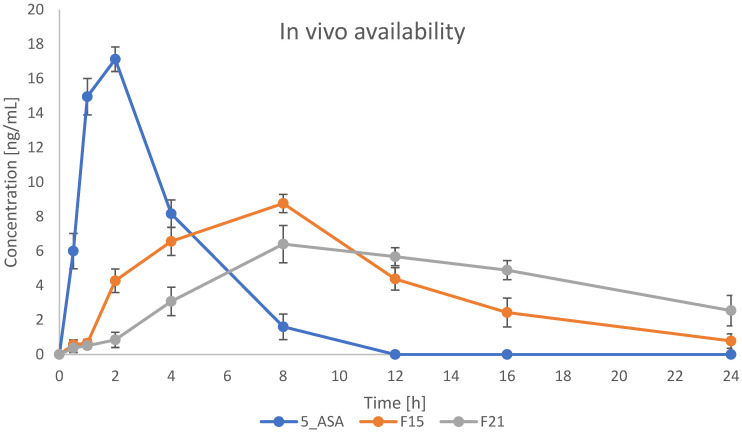
Plasma concentration-time profiles of 5-ASA in rats after administration of prepared beads and 5-ASA suspension (*n* = 4).

The most promising formulations were selected for in vivo studies. Based on all performed and previously described results, formulations containing κC were used in this experiment. Formulations F15 and F21 were indicated to evaluate the effect of GA crosslinking on bead properties in vivo. Plasma concentration-time profiles for 5-ASA are presented in Figure 15. As shown on the graph, drug plasma concentration in the case of bulk drug suspension reached the highest level (17 ng/mL) in around 2 h after administration, and then the drug was rapidly eliminated from the blood, reaching 0 ng/mL after 12 h. Prepared formulations caused the prolonged and sustained release of 5-ASA. Plasma concentration started to grow after 2 h, reaching the maximum level of 8 ng/mL and 6 ng/mL for IG and IG+GA formulations, respectively, after 6 h. A decrease in concentration was then seen more rapidly in formulation F15 than in F21. After 24 h from the administration, the concentration of 5-ASA in plasma was still around 4 ng/mL for formulation F21. It suggests that the crosslinking using GA significantly changes the behavior of the administered drug in the organism. Compared to the paper previously published, we obtained comparable results in the case of blood concentration for bulk 5-ASA suspensions. However, gellan gum and κC-based formulation resulted in changing the pharmacokinetics of the drug, providing a more prolonged manner of release and extending the time the drug stays in the body [47]. 

As it was previously published, pellets containing 5-ASA have a great potential to overperformance marketed drugs containing this anti-inflammatory agent. Sardou et al. presented layered and matrix pellets that have superior activity against ulcerative colitis (UC) on the rat model in preclinical studies and succeeded in delivering the API to the colon [48]. On the other hand, Rudolph et al. achieved a satisfactory release profile indicated as more appropriate to the profile of human ileum pH than the marketed drugs containing 5-ASA [49]. 

The presented project was planned as a pre-formulation study and did not focus on activity against inflammation. The main goal was to evaluate the influence of excipients on final properties and availability. However, a very important factor in the final activity of a drug, especially in terms of activity in distal parts of the GIT, is microbiota. The presented work was conducted on a rat model, and here it has to be stated that human microbiota slightly differs from rat microbiota, and these differences can affect how drugs are absorbed and metabolized. This can lead to different oral bioavailability of drugs in humans and rats. There are several common species as *Bacteroidetes*, *Firmicutes*, or *Proteobacteria*, but rat guts contain *Prevotella*, *Clostridiales*, *Spirochaetes*, and *Cyanobacteria*, whereas human contains more *Actinobacteria*, *Ruminococcaceae*, and *Verrucomicrobia* [50,51]. Furthermore, the expression levels of CYP3A4 and CYP3A9 differ between humans and rats, which can lead to different drug metabolism in the intestine [52]. The gut microbiota can influence the bioavailability of drugs by enzymatically transforming the drug’s structure. As Weesrma et al. presented, antibiotic-treated rats have higher levels of acetaminophen glutathione conjugates in their blood than untreated rats [53]. In the final activity, other drugs taken by patients have to be evaluated. Drugs like proton pump inhibitors (PPIs) and metformin can change the composition and function of the gut microbiome. These changes can reduce drug efficacy [51]. All the mentioned factors can influence the bioavailability of a final product and have to be evaluated at the next stages of product development.

A helpful and interesting method to evaluate the formulation distribution in the GIT is MRI imaging. As was described by Vieira et al., GG has a suitable property to be visualized via this method. Beyond enabling in situ crosslinking of the hydrogel, the ionic interaction between GG and metal ions can also be leveraged to incorporate Mn^2+^ into the hydrogel matrix for MRI-based tracking. Studies have demonstrated that Mn^2+^ binds strongly to GG, particularly at the carboxyl groups of its D-glucuronate units. Their research has shown that degradable hydrogel blends composed of methacrylated GG (GG-MA) and hyaluronic acid can be combined with Mn^2+^ to facilitate image-guided intrathecal delivery of cells. This feature may be used to observe 5-ASA-loaded beads distributed in the GIT of rats after oral administration and determine whether the formulation reached to distal parts of the GIT [54].

## 4. Conclusions

The results obtained suggest that matrices based on gellan gum and its blends with other polymers, manufactured by using ionotropic gelation and chemical crosslinking methods, can be a suitable dosage form to deliver mesalazine to the distal parts of the gastrointestinal tract. Moreover, GA affects the beads’ properties significantly, resulting in changes to the swelling behavior and release rate both in vitro and in vivo of the formulation, while not increasing toxicity. It makes it a suitable candidate for colon delivery of API for the potential treatment of bowel inflammatory diseases.

## Figures and Tables

**Table 1:** The percentage amount of ingredients in the initial mixtures used for bead manufacturing. 5-ASA—mesalazine, HEC—hydroxyethyl cellulose, κC—κ-carrageenan, HD—heat drying, FD—freeze-drying.

Formulation	Gellan	5-ASA	Tween 80	H_2_O	Additional Polymer	Crosslinking Method	Drying Method
F1	1.5	×	0.37	96.63	×	IG	HD
F2	1.0	×	0.37	96.63	HEC 0.5	IG	HD
F3	1.0	×	0.37	96.63	κC 0.5	IG	HD
F4	1.5	×	0.37	96.63	×	IG	FD
F5	1.0	×	0.37	96.63	HEC 0.5	IG	FD
F6	1.0	×	0.37	96.63	κC 0.5	IG	FD
F7	1.5	×	0.37	96.63	×	IG+GA	HD
F8	1.0	×	0.37	96.63	HEC 0.5	IG+GA	HD
F9	1.0	×	0.37	96.63	κC 0.5	IG+GA	HD
F10	1.5	×	0.37	96.63	×	IG+GA	FD
F11	1.0	×	0.37	96.63	HEC 0.5	IG+GA	FD
F12	1.0	×	0.37	96.63	κC 0.5	IG+GA	FD
F13	1.5	1.5	0.37	96.63	×	IG	HD
F14	1.0	1.5	0.37	96.63	HEC 0.5	IG	HD
F15	1.0	1.5	0.37	96.63	κC 0.5	IG	HD
F16	1.5	1.5	0.37	96.63	×	IG	FD
F17	1.0	1.5	0.37	96.63	HEC 0.5	IG	FD
F18	1.0	1.5	0.37	96.63	κC 0.5	IG	FD
F19	1.5	1.5	0.37	96.63	×	IG+GA	HD
F20	1.0	1.5	0.37	96.63	HEC 0.5	IG+GA	HD
F21	1.0	1.5	0.37	96.63	κC 0.5	IG+GA	HD
F22	1.5	1.5	0.37	96.63	×	IG+GA	FD
F23	1.0	1.5	0.37	96.63	HEC 0.5	IG+GA	FD
F24	1.0	1.5	0.37	96.63	κC 0.5	IG+GA	FD

**Table 2:** The calculated EC_50_ values for all the materials after 5 and 15 min of exposure.

Formulation	5 min	15 min	Formulation	5 min	15 min
	EC_50_	EC_50_		EC_50_	EC_50_
F1	11.98%	6.65%	F13	18.03%	9.16%
F2	6.90%	5.70%	F14	7.73%	6.25%
F3	8.20%	4.48%	F15	25.99%	12.23%
F4	4.01%	0.85%	F16	2.54%	1.09%
F5	3.46%	0.86%	F17	2.82%	1.95%
F6	6.82%	4.76%	F18	2.72%	1.94%
F7	4.93%	0.36%	F19	9.81%	4.69%
F8	3.57%	0.38%	F20	5.76%	3.75%
F9	2.74%	n/a	F21	7.37%	0.63%
F10	n/a	n/a	F22	3.73%	0.53%
F11	n/a	n/a	F23	n/a	n/a
F12	n/a	n/a	F24	0.70%	n/a

n/a—the EC_50_ value could not be calculated correctly due to the very high toxicity of the samples in the tested concentration range.

## Data Availability

The original contributions presented in this study are included in the article. Further inquiries can be directed to the corresponding author(s).
